# Identification of Methylation Immune Subtypes and Establishment of a Prognostic Signature for Gliomas Using Immune-Related Genes

**DOI:** 10.3389/fimmu.2021.737650

**Published:** 2021-11-04

**Authors:** Zhengang Hu, Hao Zhang, Fan Fan, Zeyu Wang, Jiahao Xu, Yunying Huang, Ziyu Dai, Hui Cao, Xun Zhang, Zhixiong Liu, Quan Cheng

**Affiliations:** ^1^ Department of Neurosurgery, Xiangya Hospital, Central South University, Changsha, China; ^2^ Department of Psychiatry, The Second People’s Hospital of Hunan Province, The Hospital of Hunan University of Chinese Medicine, Changsha, China; ^3^ National Clinical Research Center for Geriatric Disorders, Xiangya Hospital, Central South University, Changsha, China; ^4^ Department of Clinical Pharmacology, Xiangya Hospital, Central South University, Changsha, China

**Keywords:** glioma, methylation, immune subtype, prognostic signature, pan-cancer

## Abstract

DNA methylation patterns are essential in understanding carcinogenesis. However, the relationship between DNA methylation and the immune process has not been clearly established—this study aimed at elucidating the interaction between glioma and DNA methylation, consolidating glioma classification and prognosis. A total of 2,483 immune-related genes and 24,556 corresponding immune-related methylation probes were identified. From the Cancer Genome Atlas (TCGA) glioma cohort, a total of 683 methylation samples were stratified into two different clusters using unsupervised clustering, and eight types of other cancer samples from the TCGA database were shown to exhibit excellent distributions. A total of 3,562 differentially methylated probes (DMPs) were selected and used for machine learning. A five-probe signature was established to evaluate the prognosis of glioma as well as the potential benefits of radiotherapy and Procarbazine, CCNU, Vincristine (PCV) treatment. Other prognostic clinical models, such as nomogram and decision tree, were also evaluated. Our findings confirmed the interactions between immune-related methylation patterns and glioma. This novel approach for cancer molecular characterization and prognosis should be validated in further studies.

## Introduction

Glioma, which develops from glial cells, is the most common type of primary central nervous system tumor (1). Therapeutic options for glioma include surgical resection, radiation, and chemotherapy. However, the overall survival (OS) time continues to be low.

Molecular markers have been shown to be efficient in predicting prognosis, including mutational status, mRNA expression, and DNA methylation. Several molecular markers (MGMT, 1p/19q, IDH, EGFR, p53, PI3K, Rb, and RAF) have been successfully used for the classification and prediction of prognosis (2). The immune system plays a crucial role in antineoplasms (3). Apart from cancer cells, the tumor microenvironment (TME) contains various non-carcinogenic cell types, including endothelial cells, pericytes, and fibroblasts (4). Characteristically, as tumor grade increases, patients present with heightened levels of immunosuppression (5). A typical human immune response comprises humoral and cell-mediated reactions that shield the body against foreign bodies, including microscopic organisms, infections, and tumors. To prevent autoimmune responses, various immune cells such as regulatory T cells, monocytes, and neutrophils are used to suppress inflammation and maintain self-tolerance (6–8). Given that all options for managing glioma, temozolomide chemotherapy, radiotherapy, and corticosteroids, exhibit immunosuppressive effects, therapeutic options should confer less reduced side effects and clinical complications (3).

DNA methylation patterns are now perceived as heritable alterations in gene expression and are highly involved in carcinogenesis (9). A minimally invasive biopsy can improve diagnosis, treatment measures, and prediction of prognosis in cancer. Peculiar DNA methylation patterns influence critical genes of carcinogenesis and progression and inhibit some tumor suppressor genes (10). Therefore, understanding epigenetic changes can improve the characterization of malignancy patterns to predict treatment responses and prognosis (11). Even though DNA methylation patterns of specific genes have been reported, the scope of immune-related gene methylation patterns on glioma and other tumors has not been clearly established.

This study aims to classify and predict glioma outcomes by concentrating on the interaction between the immune process and methylation profile. We sought to identify immune-related probes and build an immune-related cluster using unsupervised clustering analysis. Moreover, we performed pan-cancer analysis to identify a pan-cancer immune pattern to subdivide cancer among patients. Then, we established an immune-related prognostic signature, a clinical decision tree model, and a nomogram to inform on customized therapy and prognosis.

## Materials and Methods

### Glioma Patient Data

**Figure 1:**
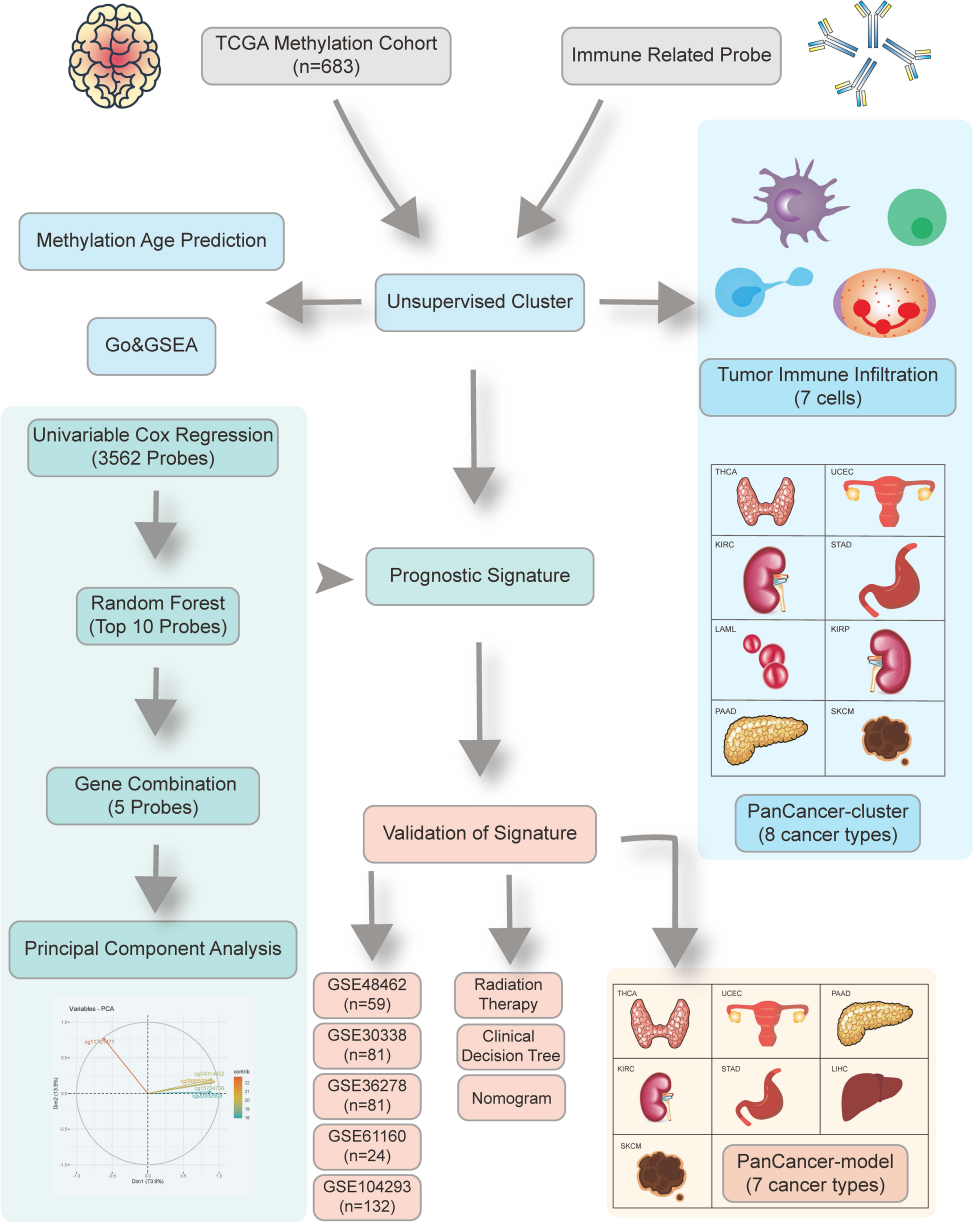
Overview of the study design. First, we identified 24,556 candidate immune-related methylation probes. Then unsupervised cluster was used in a 683 TCGA glioma methylation cohort. A five-CpG-based prognostic signature was established and validated in five independent GEO datasets.

The schematic presentation of the study is shown in **Figure 1**.

This study involved a total of 683 glioma samples from TCGA databases and were retrieved from UCSC Xena TCGA database (https://tcga.xenahubs.net). RNA-seq data of 587 glioma samples were downloaded from the TCGA database (http://cancergenome.nih.gov/). Correlative clinical traits and molecular features were also retrieved from the TCGA database. Pan-cancer data atlas were downloaded from the UCSC Xena TCGA database. The Gene Expression Omnibus (GEO) dataset (GSE48462, GSE61160, GSE36278, GSE30338, and GSE104293) was obtained from the GEO database (https://www.ncbi.nlm.nih.gov/geo/). The platform of the methylation dataset was Illumina HumanMethylation450 BeadChip (GPL13534).

### Construction of Immune-Related Probes

Inclusive immune-related genes (2,483 genes) were retrieved from the ImmPort database (version: July 2020) (https://www.immport.org/shared/genelists). Corresponding probes in 450k chipset annotation file (GPL13534) were matched, after which 24,556 immune-related probes participating involved in the immune process were eventually selected.

### Establishment of Immune-Related Clusters Based on Immune-Related Probes Using Consensus Clustering

We used the R package “ConsensusClusterPlus” to perform unsupervised clustering analysis (12). A total of 683 glioma samples accompanied by 24,556 immune-related probes were divided into different methylation subtypes using K-means and Euclidean distance as the baseline clustering algorithm and the similarity measure, respectively. After 1,000 iterations, the optimal k value was selected using the Proportion of Ambiguous Clustering (PAC) measure, which had the lowest PAC.

### Identification of Genome-Wide Methylation Probes

R package “ChAMP” was used to preprocess and analyze the Illumina Infinium 450k DNA methylation array (13). Probes with missing values greater than 20% were deleted. Then probes were filtered by “champ.filter” to remove those with p > 0.01, bead count <3, noCG start, Single Nucleotide Polymorphism (SNP) (14), multihit, and those targeting the X and Y chromosomes. Other missing values were imputed by k-nearest neighbor (KNN) imputation algorithm. The normalized beta value matrix was established by “champ.norm” using the Beta MIxture Quantile dilation (BMIQ) method (15). Differential methylation probes were detected using “limma” package to calculate the p-value for differential methylation using a linear model (16). The Benjamini and Hochberg false discovery rate method was used to reduce the false positive rate (FDR) when applying multiple comparisons. Delta beta means the average discrepancy of beta value between two different groups. Adjusted p<0.05 and delta beta>0.2 were selected as cutoff values for detecting DMPs. Simultaneously, differentially expressed genes were identified by “limma” package with 0.5 logFC cutoff and p value <0.05 (17).

### Functional Annotations

Gene ontology (GO) analysis of the methylation profile was performed using “clusterProfiler” package (18). We used the Benjamini and Hochberg (BH) method to determine the adjusted p-values, and pathway in which FDR values were under 0.05 was chosen.

The Empirical Bayes Gene Set Enrichment Analysis (ebGSEA) was used to identify the exact enriched genes for specific biological terms or pathways (19). This method can be used to independently perform GSEA, regardless of DMPs or differentially methylated regions. Moreover, it can be used to identify significant genes, with the bias of inequality of CpG number corrected for each gene. GSEA of the TCGA RNA-seq cohort was estimated using the “clusterProfiler” package (18).

### Profiling DNA Methylation Age and Tumor Infiltration

Based on Horvath’s clock model, we used the “agep” method in the “wateRmelon” package to predict DNA methylation age from normalized methylation β values (19).

Tumor-infiltrating immune analysis of the methylation set was performed using the “EpiDish” package (20). We identified proportions of *a priori* known cell subtypes present in a sample representing a mixture of such cell types. FractionS of eight cell subtypes (B-cells, CD4+ T-cells, CD8+ T-cells, NK-cells, Monocytes, Neutrophils, Eosinophils, Neutrophils, and Eosinophils) were estimated using the Robust Partial Correlations (RPC) method and whole blood subtypes reference (21). Meanwhile, the tumor-infiltrating immune analysis of transcriptome was evaluated using the CIBERSORT algorithm (22). A total of 22 immune cells are calculated in 1,000 times permutations.

### Construction and Validation of a Prognostic Model

Univariate Cox regression analyses were performed using the “survival” package with a 0.05 p-value cutoff. Then Random Forest algorithm was used to rank the top 10 methylation probes that contribute to prediction. Therefore, the “randomForestSRC” package was used, and the number of trees was 1,000. Kaplan-Meier analysis was performed to establish the best combination of the 1,023 participant model. After selecting the best combination model, exact coefficients of each probe were determined by the Principal Component Analysis (PCA) method. FactoMineR and “factoextra” package contributed to applying PCA (23). The risk score for each patient after the prognostic value of each gene signature score was obtained using the formula: risk score = SPC1i - SPC1j, where *i* represented the expression of genes with HR>1, and *j* the expression of genes with HR<1. Risk score = 10.52*cg11701471-22.14*cg04314652-21.95*cg04314652-21.34*cg20332504-24.02*cg08985333. To make the cutoff value more accurate, we employed the survival cutoff method to determine the optimal cutpoint that corresponds to the most significant relation with overall survival. The cutoff point of the high- and low-risk group in the TCGA cohort was −33.57. Receiver operating characteristic (ROC) analysis was performed using “timeROC” package (24). Clinical benefits were estimated using decision curve analysis and were established by R package “rmda” (25). Five GEO datasets (GSE48462, GSE61160, GSE36278, GSE30338, GSE104293) were chosen as validation set.

### Identification of Clinical Associated Risk Model

A nomogram and corresponding calibration curve were established using the “rms” in R package. Univariate and multivariate Cox proportional hazard analyses were performed based on the risk score and clinical factors. After multivariate Cox proportional hazard analysis, factors with p<0.05 were chosen to establish the nomogram. Forest plots were constructed using the “forestplot” and “ggforest” packages. Recursive partitioning analysis was performed using the “rpart” and “rattle” packages to construct a survival-related decision tree and stratify the prognostic risk. Concordance index (C-index) and ROC analysis were used to evaluate the predictive values of survival among the different models.

### Prediction of Radiotherapeutic and Chemotherapeutic Responses

The TCGA GBM cohort was used to predict patient responses to radiotherapy. The GSE48462 dataset, which was an Anaplastic Oligodendrogliomas and Oligoastrocytomas cohort treated with RT or RT/PCV, was used for the prediction of patient responses to PCV therapy (26). Samples with Illumina HumanMethylation27k BeadChip were excluded.

### Statistical Analysis

Pearson correlation analyses were performed to establish correlation coefficients. The chi-square test was used to analyze count data, while the Wilcoxon rank-sum test was used to analyze continuous data. Kaplan-Meier survival analysis with log-rank test was used to assess survival differences between different groups. Data were depicted using the “ggplot2” package. The cutoff between high-risk and low-risk was determined by “surv_cutpoint” function in “survminer” package, and all the survival curves were visualized by the “survminer” package. Heatmaps are presented using the “pheatmap” package. All statistical analyses were performed by R software. p<0.05 was considered statistically significant.

## Results

### Identification of Immune-Related Clusters

**Figure 2:**
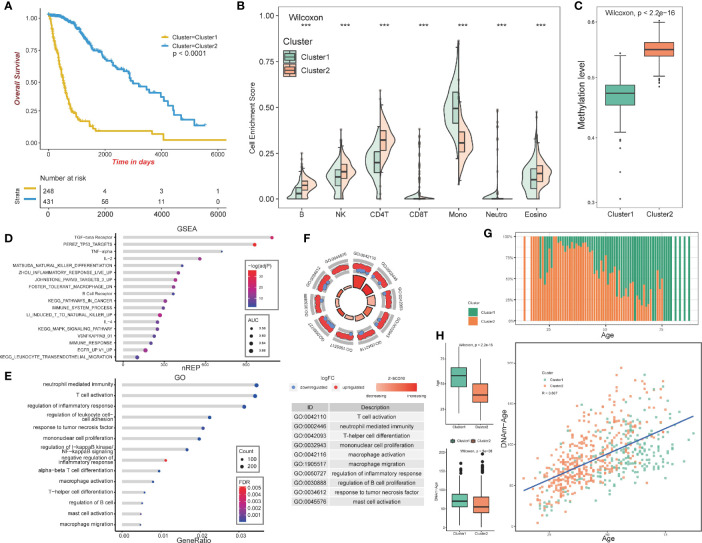
Construction and analysis of consensus cluster. **(A)** Kaplan–Meier survival analysis of the two clusters showing clear separation. **(B)** Tumor-infiltrating immune analysis of the two clusters based on TCGA methylation set. **(C)** Methylation levels of the two clusters. **(D)** ebGSEA result of the two clusters. **(E, F)** GO functional enrichment analysis of the two clusters. **(G)** Age distribution of the two clusters. **(H)** Box plot showing the age and DNA methylation age difference between the two clusters, Scatter plot revealing correlations of the DNA methylation age-age correlation coefficients between cluster1 and cluster2. ***p <0.001.

The overall design of our study is shown in **Figure 1**. We aimed at identifying immune-related clusters in the TCGA glioma cohort (n=683) by utilizing the 24,556 immune-related methylation probes using the ConsensusClusterPlus package (**Table S1**). Clinical information of the TCGA glioma cohort is shown in **Table 1**. Clustering results are presented in a cumulative distribution function (CDF) plot and a delta area plot (**Figures S1A, B**). The CDF plot and delta area plot revealed that the optimal k value for stable distribution was 2. We selected k=2 as the epitome number of clusters, and the heatmap of the consensus matrix showed satisfactory discrimination (**Figure S1C**). Kaplan-Meier survival analysis revealed that cluster2 had a more favorable prognosis (p<0.0001; **Figure 2A**). To elucidate on the immune microenvironment status between the two clusters, tumor-infiltrating immune analysis was performed in both the TCGA methylation set and the expression set (n=587). In the methylation set, we used the EpiDISH package to evaluate differences in seven immune cells, including B cells, Natural killer (NK) cells, CD4T cells, CD8T cells, monocytes, neutrophils, and eosinocytes. Levels of all the seven immune cells were significantly different (p<0.001), and cluster2 exhibited a higher enrichment score of B cells, NK cells, CD4T cells, and eosinocytes. In contrast, cluster1 exhibited a higher proportion of CD8+T cells, monocytes, and neutrophils (**Figures 2B**, **S1F**). The immune microenvironment difference in the TCGA RNA-seq cohort using the CIBERSORT algorithm (**Figures S1G**). Methylation levels between clusters 1 and 2 were significantly different (p<2.2e-16), with a higher methylation level in cluster2 (**Figure 2C**). Since the different methylation statuses may contribute to biological changes, ebGSEA analysis was performed. It was found that corresponding genes of DMPs were concentrated in the cytokine-receptor pathways such as TGF−beta Receptor (TGF-β), TP53, tumor necrosis factor-alpha (TNFα), and interleukin-2(IL-2), which implied a strong relationship between glioma tissues and inflammatory responses (**Figure 2D**). Furthermore, GO functional enrichment analysis revealed that corresponding genes of DMPs were enriched in pathways involving neutrophils, T cells, B cells, macrophages, and mast cell signaling pathways, implying that the two clusters had vital differences regarding immune status (**Figures 2E, F**).

**Table 1:** Clinical and genetic characteristics of patients after clustering.

	GBM		LGG		TOTAL	
	Cluster1	Cluster2	Cluster1	Cluster2	Cluster1	Cluster2
	(N=146)	(N=7)	(N=104)	(N=426)	(N=250)	(N=433)
**Gender**						
Female	60 (41%)	1 (14%)	45 (4%)	163 (38%)	105 (42%)	164 (38%)
Male	80 (55%)	5 (71%)	49 (47%)	211 (50%)	129 (52%)	216 (50%)
Missing	6 (4.1%)	1 (14.3%)	10 (9.6%)	52 (12.2%)	16 (6.4%)	53 (12.2%)
**Age (years)**						
<52	31 (21%)	5 (71%)	42 (40%)	292 (69%)	73 (29%)	297 (69%)
>66	48 (33%)	0 (0%)	14 (13%)	17 (4%)	62 (25%)	17 (4%)
52–66	61 (42%)	1 (14%)	38 (37%)	65 (15%)	99 (40%)	66 (15%)
Missing	6 (4.1%)	1 (14.3%)	10 (9.6%)	52 (12.2%)	16 (6.4%)	53 (12.2%)
**IDH**						
Mutant	2 (1%)	7 (100%)	8 (8%)	423 (99%)	10 (4%)	430 (99%)
WT	135 (92%)	0 (0%)	96 (92%)	0 (0%)	231 (92%)	0 (0%)
Missing	9 (6.2%)	0 (0%)	0 (0%)	3 (0.7%)	9 (3.6%)	3 (0.7%)
**pq**						
Codel	0 (0%)	0 (0%)	1 (1%)	171 (40%)	1 (0%)	171 (39%)
Non-codel	143 (98%)	7 (100%)	103 (99%)	255 (60%)	246 (98%)	262 (61%)
Missing	3 (2.1%)	0 (0%)	0 (0%)	0 (0%)	3 (1.2%)	0 (0%)
**MGMT**						
Methylated	56 (38%)	6 (86%)	40 (38%)	397 (93%)	96 (38%)	403 (93%)
Unmethylated	79 (54%)	0 (0%)	64 (62%)	29 (7%)	143 (57%)	29 (7%)
Missing	11 (7.5%)	1 (14.3%)	0 (0%)	0 (0%)	11 (4.4%)	1 (0.2%)
**Radiation therapy**						
Yes	108 (74%)	7 (100%)	70 (67%)	226 (53%)	178 (71%)	233 (54%)
No	17 (12%)	0 (0%)	21 (20%)	164 (38%)	38 (15%)	164 (38%)
Missing	21 (14.4%)	0 (0%)	13 (12.5%)	35 (8.2%)	34 (13.6%)	35 (8.1%)

In summary, the GO functional enrichment analysis and the association between EpiDISH and CIBERSORT results of the immune infiltrating microenvironment indicated the abnormal immune infiltrations in cluster1 might serve as prognostic indicators for strong inflammatory reactions and poor overall outcomes.

### Analysis of DNA Methylation Age and Clinical and Molecular Feature

The patients in cluster1 trended to be older than cluster2 (**Figure 2G**). We identified the DNAm ages in the TCGA methylation cohort (n=683) using the “wateRmelon” package and compared them with their chronological ages. Chronological ages (Wilcoxon, p < 2.2e-16) and DNAm ages (Wilcoxon, p = 5e−06) were found to be higher in cluster1 than in cluster2. Furthermore, the correlations between chronological ages and DNAm ages were significantly high (Pearson, R=0.607), indicating that epigenetic age analysis might contribute to glioma patients’ prognostic prediction (**Figure 2H**).

Moreover, we evaluated gender, histology, IDH, 1p/19q, and MGMT distributions between the two clusters. Histology, IDH, 1p/19q, and MGMT were found to be significantly distributed (Pearson’s chi-squared test, p<0.001). However, no evidence was found for gender associations between the two clusters (**Figure S2A**).

We also employed the survival differences considering the types of gliomas with the cluster. In details, cluster1 enjoyed worse prognosis than cluster2 in GBM cohort (p = 0.013) and in LGG cohort (p < 2e-16). LGG cohort possessed higher survival possibilities than GBM cohort in cluster1 (p < 0.0001) and in cluster2 (p = 0.048, **Figure S2B**).

These results suggest that there is a difference in epigenetic age and clinical traits.

### Differentially Methylated Probes and Differentially Expressed Genes in the Two Clusters

**Figure 3:**
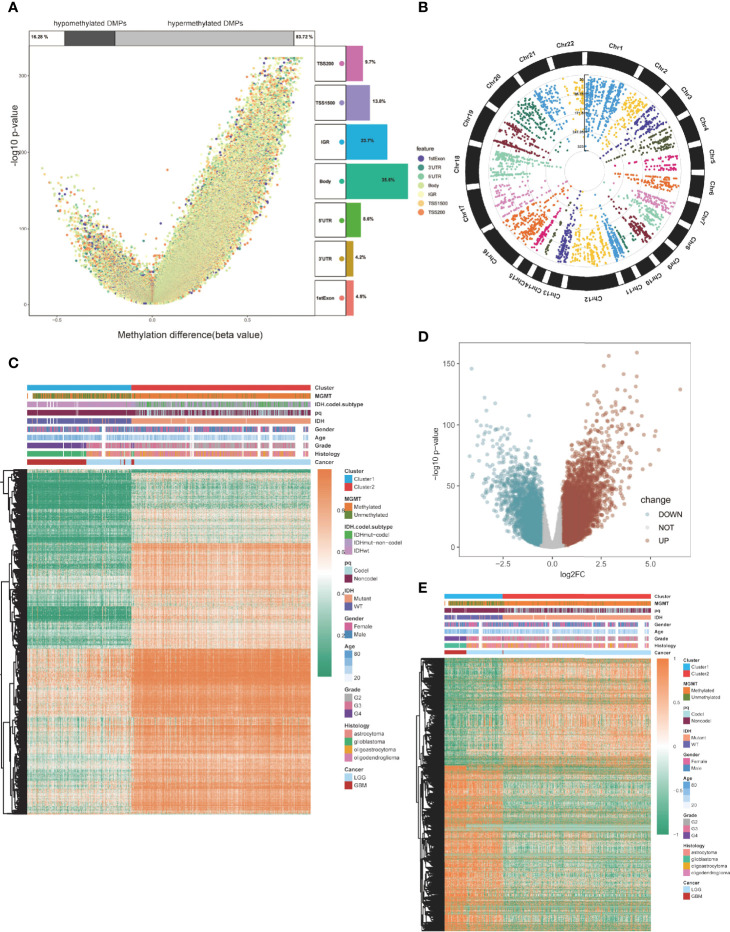
Global DMPs and DEGs of the two clusters. **(A)** Volcano plot. Proportions of hypermethylated and hypomethylated DMPs are shown on top. The distribution of DMPs’ feature is shown on the right. **(B)** The manhattan plot of the DMPs. **(C)** Heatmap of the two clusters based on the TCGA methylation set. High methylation, orange; low methylation, green. **(D)** Volcano plot of the DEGs. **(E)** Heatmap of the twp clusters based on the TCGA RNA-seq cohort. High expression, red; low expression, green.

Then, we compared global patterns between the two clusters in the TCGA glioma methylation and expression sets. In the methylation set, package “ChAMP” was used, and 56886 DMPs were identified from Illumina Infinium 450k DNA methylation data of 683 samples according to the cutoff of Delta beta>0.2 and p value<10^-5. Compared to cluster1, 55,186 DMPs were upregulated, while 1,700 DMPs were downregulated (**Figure 3A**). At most, 5,964 DMPs were located on chromosome 1, and at least 492 DMPs were located on chromosome 21 (**Figure 3B**). The overview of unsupervised clustering analysis of genome-wide DMPs is shown in the heatmap. Clinical and demographic features, including MGMT, IDH with codel subtype, 1p/19q, IDH, sex, age, grade, histology, and cancer type, are also shown in the heatmap (**Figure 3C**).

Differentially expressed genes (DEGs) were identified using the “limma” package in the expression section, and genes with log2FC > 0.5 and p < 0.05 were selected (**Figure 3D**). Global DEGs and clinical characteristics were visualized by heatmap (**Figure 3E**).

### Pan-Cancer Analysis of Immune-Related Methylation Probes

**Figure 4:**
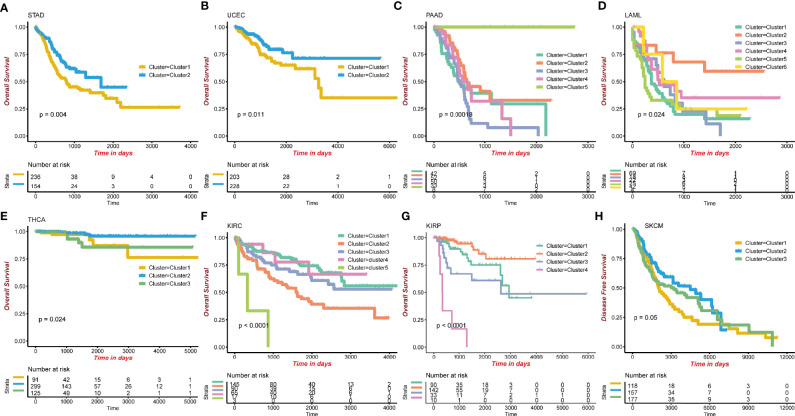
Eight cancer types showing significant outcomes in TCGA pan-cancer survival analysis using consensus cluster. **(A)** STAD, Stomach adenocarcinoma. **(B)** UCEC, Uterine Corpus Endometrial Carcinoma. **(C)** PAAD, Pancreatic adenocarcinoma. **(D)** LAML, Acute Myeloid Leukemia. **(E)** THCA, Thyroid carcinoma. **(F)** KIRC, Kidney renal clear cell carcinoma. **(G)** KIRP, Kidney renal papillary cell carcinoma. **(H)** SKCM, Skin Cutaneous Melanoma.

To evaluate the methylation levels of immune-related probes in other types of TCGA cancers, we applied the unsupervised consensus clustering method to other 31 cancer types in TCGA. After selecting optimal *k*, Kaplan-Meier survival analysis was performed, with the 0.05 significant p-value cutoff. Finally, eight cancer types, including Stomach adenocarcinoma (STAD) (p=0.004), Uterine corpus endometrial carcinoma (UCEC) (p=0.011), Pancreatic adenocarcinoma (PAAD) (p=0.00018), Acute myeloid leukemia (LAML) (p=0.024), Thyroid carcinoma (THCA) (p=0.024), Kidney renal clear cell carcinoma (KIRC) (p<0.0001), Kidney renal papillary cell carcinoma (KIRP) (p<0.0001), and Skin cutaneous melanoma (SKCM), were selected (p=0.05; **Figures 4A–H**). The p-values of pairwise comparison were also identified (**Table S2**). This methylation pattern indicates that further evaluation is required.

### Construction of a Prognostic DNA Methylation Signature for Glioma

**Figure 5:**
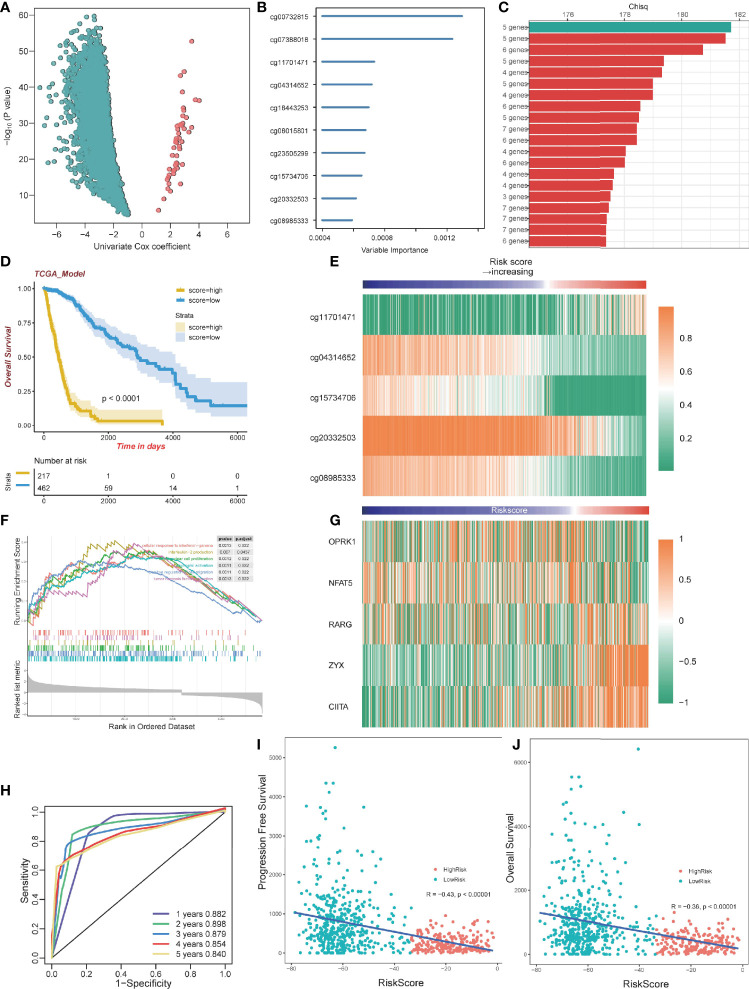
Construction of a prognostic model. **(A)** Volcano plot showing the selected probes by univariate Cox regression analysis. The horizontal axis represents log2(HR) **(B)** Variable importance of 10 probes determined by random survival forest analysis. **(C)** Top 20 combinations of signatures were selected after Kaplan–Meier analysis according to the chi-square value. A five-probe signature with the highest chi-square was identified. All 20 signatures had a significant p-value Kaplan–Meier analysis. **(D)** Kaplan–Meier analysis of the high-risk and low-risk group. **(E)** Heatmap of the five selected methylation probes sorted by risk score. **(F)** GSEA plots for the enrichment of immunogenic and oncogenic signaling pathways from the GO. **(G)** Heatmap of gene expression corresponding to the selected five probes sorted by risk score. **(H)** ROC curve of the TCGA dataset. **(I)** The correlation between PFS time and risk score. **(J)** The correlation between OS time and risk score.

To select optimal DNAm markers for glioma patients, we used 24,556 immune-related probes for differential methylation analysis of 683 TCGA tumor samples by adopting the standard of Delta beta>0.2 and p value<10^-5. A total of 3,562 probes were selected. Then univariate Cox regression analysis was performed with overall survival data, and all 3,562 markers were selected (**Figure 5A**; **Table S3**). After univariate Cox regression analysis, the 3,562 probes were put into machine learning algorithms, using Random Forest with overall survival profile, and 10 probes were selected (cg20332503, cg15734706, cg23505299, cg08015801, cg18443253, cg04314652, cg11701471, cg07388018, cg00732815, cg08985333) (**Figures 5B**, **S1D**). The 10 probes could form 1,023 combinations (**Table S4**), and Kaplan-Meier analysis was performed to establish the best probe combination. First, we contrasted their Log-rank p-values. However, the top 10 signature p values turned out to be close to 0 infinitely, which meant that their overall survival conditions were significantly distinct but not comparable. Therefore, we compared chi-squares (chisq) of each model and a five-probe signature (cg11701471, cg04314652, cg08985333, cg15734706, cg20332503) with the highest chi-square was selected finally (**Figure 5C**). Profile of the five methylation probes was given (**Table 2**). Then, coefficients of the signatures were determined by PCA (**Figure S1E**).

**Table 2:** Five selected methylation probes.

	Chr	Mapinfo	Strand	Type	Gene	Feature	Cgi	UCSC_CpG_Islands_Name
**cg11701471**	8	54164051	R	I	***OPRK1***	5’UTR	island	chr8:54163303-54164443
**cg04314652**	16	69597925	F	II	***NFAT5***	TSS1500	shore	chr16:69599437-69600736
**cg15734706**	12	53614080	F	I	***RARG***	1stExon	island	chr12:53613716-53615103
**cg20332503**	7	143081287	R	II	***ZYX***	Body	shelf	chr7:143077469-143079169
**cg08985333**	16	10970960	F	II	***CIITA***	TSS200	shore	chr16:10972782-10973305

5’UTR, 5′ untranslated region; TSS200, −200 base pairs upstream of the Transcription Start Site; TSS1500, between −200 and −1,500 base pairs upstream of the Transcription Start Site.

### Evaluation of the Signature and Pan-Cancer Analysis

By dividing the TCGA glioma cohort into high-risk groups (n=267) and low-risk groups (n=462) using the survival cutoff, high‐risk patients were short‐lived compared to low-risk patients (log-rank test p<0.0001) (**Figure 5D**). Beta values of the selected probes are presented in the heatmap (**Figure 5E**). We also verified the RNA-seq profiles of the five selected probes. Gene Set Enrichment Analysis (GSEA) revealed that cluster1 exhibited significant immune processes (**Figure 5F**). The RNA-seq data of the corresponding genes were also presented in the heatmap (**Figure 5G**). The impact of DNA methylation on gene expression in glioma was also evaluated. Three of five corresponding genes (cg15734706 and RARG, cg20332503 and ZYX, cg08985333 and CIITA) were considered significantly different between the high-risk group and low-risk group, and they share the same pattern that they were both hypomethylated and upregulated genes (**Figure S3A–E**). We further excavated the interaction between DNA methylation and gene expression by conducting Spearman’s rank correlation analysis. It showed that cg20332503, cg15734706, and cg08985333 exhibited negative correlation, while cg20332503 (ρ=−0.5) and cg08985333 (ρ=−0.6) showed extremely high correlation (**Figure S3F–J**).

Then, we performed ROC to evaluate the predictive value of the signature, and we found that the area under curve (AUC) values of 1 to 5 years were all higher than 0.8, with the highest AUC (0.898) at 2 years (**Figure 5H**). Interestingly, there was a negative correlation between Progression-Free Survival (PFS) and risk score (Pearson, R=−0.43, p<0.00001; **Figure 5I**). The overall survival exhibited the same pattern with risk score (Pearson, R=−0.36, p<0.00001; **Figure 5J**), implying that patients with lower risk scores have better survival outcomes.

Then, we validated the performance of the signature using pan-cancer analysis. We downloaded the TCGA Illumina Infinium 450k DNA methylation pan-cancer data from UCSC Xena and calculated the risk scores. Using survival cutoff, they were distributed into high-risk groups and low-risk groups. After the Kaplan-Meier survival test, seven of 31 cancer types were selected: Skin cutaneous melanoma (SKCM) (p=0.00095), Pancreatic adenocarcinoma (PAAD) (p=0.00015), Liver hepatocellular carcinoma (LIHC) (p=0.00013), Thyroid carcinoma (THCA) (p=0.029), Kidney renal clear cell carcinoma (KIRC) (p<0.0001), Uterine corpus endometrial carcinoma (UCEC) (p=0.0071), and Stomach adenocarcinoma (STAD) (p=0.014) (**Figures S2C–I**). Pan-cancer analysis revealed a satisfactory prognostic value in glioma and the other seven cancer types.

### Validation of the Signature and Its Functional Enrichment

**Figure 6:**
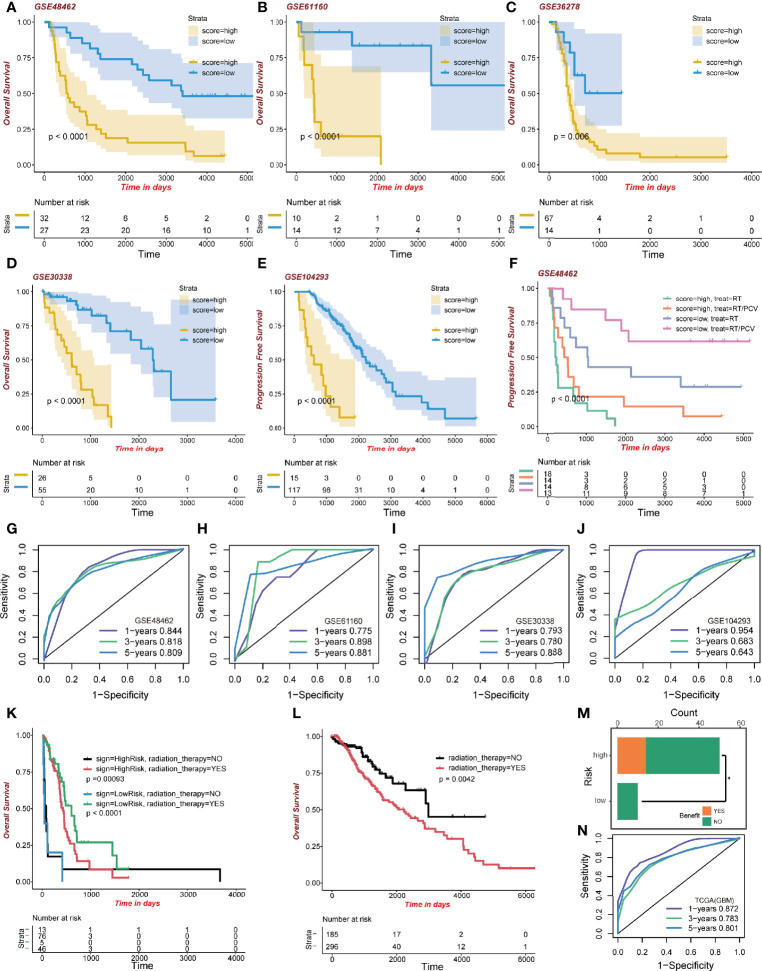
Validation of the signature. **(A–E)** Kaplan-Meier curve of high-risk and low-risk score groups in the GEO cohort. **(F)** Kaplan-Meier survival analysis of different treatments on the GSE48462 dataset. Both high-risk and low-risk groups benefited from PCV. **(G–J)** ROC curve of the GEO dataset. **(K)** Kaplan-Meier curve of radiation therapy in the high- and low-risk GBM dataset group of TCGA. **(L)** Kaplan-Meier curve of radiation therapy in the LGG dataset group of TCGA. **(M)** Benefit from RT/PCV treatment in the GSE48462 dataset. **(N)** ROC curve of the GBM dataset group in TCGA.

To confirm the performance of the signature, five GEO datasets of glioma were formed as the validation groups; they were GSE48462 (n=59; p<0.0001), GSE61160 (n=24; p<0.0001), GSE36278 (n=81; p=0.006), GSE30338 (n=81; p<0.0001), and GSE104293 (n=132; p<0.0001). High-risk group patients exhibited lower survival outcomes than low-risk group patients (**Figures 6A–E**). Moreover, the ROC curve showed a competent accuracy in each validation set (**Figures 6G–J**).

Then we performed the functional enrichment analysis of the high-risk and low-risk groups. Both GO enrichment (**Figure S2J**) and ebGSEA (**Figure S2K**) pathways were related to the immune process and may participate in glioma development.

### GBM Patients Present Favorable Clinical Benefits to Radiotherapy

What is interesting regarding data of the training set is that GBM patients, regardless of risk scores, benefited from radiotherapy. The GBM high-risk group patients who had been administered with radiotherapy exhibited better survival outcomes (483 days median OS time) than those who did not (64 days median OS time; p=0.00093; **Figure 6K**). Moreover, the low-risk GBM group exhibited some clinical benefits from radiotherapy (579 days median OS time than 19 days median OS time, p<0.0001; **Figure 6K**). However, in the LGG group, patients receiving radiotherapy exhibited worse clinical outcomes (2,235 days median OS time than 2,988 days median OS time, p=0.0042; **Figure 6L**). We evaluated the model’s predictive ability in the GBM cohort by ROC analysis (**Figure 6N**). These results revealed better radiotherapeutic outcomes.

### High-Risk Group Patients Benefitted From RT/PCV Treatment

After validating the GSE48462 cohort as the glioma validation set, we evaluated the prognostic outcomes of radiotherapy and Procarbazine, CCNU, and Vincristine (RT/PCV) treatment. We found that both high-risk and low-risk group patients treated with RT/PCV had better PFS time than those treated with RT alone (p<0.0001; **Figure 6F**). Moreover, 14 (28%) high-risk patients benefited from RT/PCV treatment, 36 (72%) high-risk patients did not benefit from RT/PCV treatment, while 9 (100%) low-risk patients failed to benefit from RT/PCV treatment. There was a significant difference between the two groups (Chi-square test p<0.05), implying that the high-risk group rather than the low-risk group exhibited favorable outcomes from RT/PCV treatment (**Figure 6M**).

### Construction of Clinical-Related Models to Precisely Demonstrate Risk Stratification in Glioma Patients

**Figure 7:**
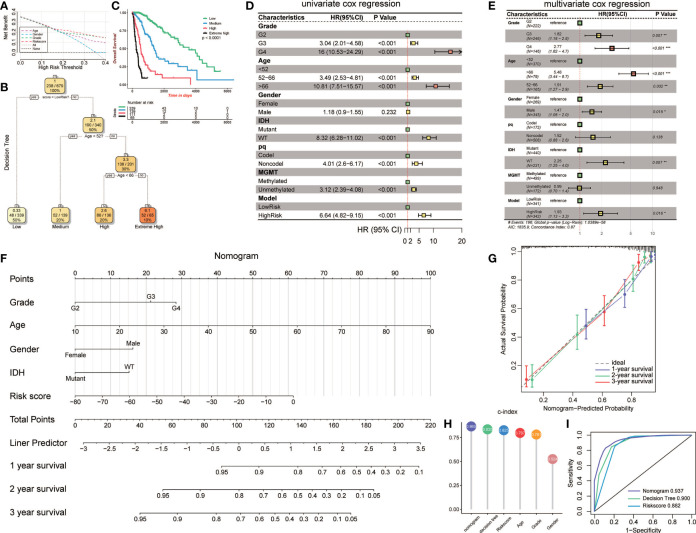
Construction and validation of the clinic-associated model. **(A)** Decision curve analysis (DCA) for age, gender, grade, and risk score, revealing the risk score revealed better survival outcomes than the other variables. **(B)** Patients with risk score and age were used to set up a detailed glioma risk stratification. **(C)** Kaplan-Meier survival analysis of the four precise risk subtypes in the TCGA dataset. **(D)** Forest plot of the univariate Cox proportional-hazards model survival analysis of various parameters. **(E)** Forest plot of multivariate Cox proportional-hazards model survival analysis of various parameters. **(F)** Details of the nomogram. **(G)** Calibration curve showing a high accuracy of the nomogram. **(H)** Comparison of the predictive power of multiple models. **(I)** ROC curve of the nomogram, decision tree, and risk score model in 1 year. HR, hazard ratio; CI, confidence interval. *p<0.05; **p<0.01; ***p <0.001.

To validate the parameters’ predictive value, we used decision curve analysis (DCA), and the risk score enjoyed the highest net benefit compared with age, gender, and grade, indicating that the risk score of models can be used as the main decision tree parameter (**Figure 7A**). Then, a total of 679 patients with detailed clinical information, including histology, grade, age, gender, status of IDH, status of pq, status of MGMT, and risk score, were selected for recursive partitioning analysis with an attempt to establish a detailed and maneuverable clinical-related model. Age and risk scores were chosen to finally establish a decision tree model. We selected two as the number of splits to prune our decision tree model since it had a relatively simple decision as well as a comparatively low standard deviation. Four risk subgroups were established based on whether the risk score was “high” or “low,” together with age distribution. The low-risk group was identified when the methylation model’s risk score was low regardless of age. The medium-risk group was identified when the risk score was high and the patient ages were lower than 52. Patients with high risk scores and aged between 52 and 66 years old were considered the high-risk group, while patients that were older than 66 years old were placed in the extremely high-risk group (**Figure 7B**). The four-class risk stratification suffered significantly different overall survival outcomes (p<0.0001, **Figure 7C**).

To establish an advanced prognostic evaluation model, we used univariate Cox regression analysis. Several parameters were passed through to the algorithm, and almost all variables fitted well (p<0.001) except for gender (HR=1.118, p=0.232; **Figure 7D**, **Table 3**). The multivariable Cox regression showed that grade, age, gender, IDH, and model were highly significant independent variables. In contrast, pq and MGMT did not fit well (**Figure 7E**). Therefore, we established a nomogram combined with risk scores and other significant parameters using the multivariate Cox regression (**Figure 7F**).

**Table 3:** Univariate Cox regression of clinical features.

Variable	Coef	HR	z	P	CI (lower)	CI (upper)
Histology	−0.27172	0.76207	−4.90692	9.25E-07	0.683692	0.849434
Grade	1.453439	4.277799	13.59881	4.07E-42	3.469318	5.274686
Age	0.068908	1.071338	12.81726	1.31E-37	1.060108	1.082686
Gender	0.165048	1.17945	1.19585	0.231755	0.899909	1.545825
IDH	2.11895	8.322394	14.78035	1.96E-49	6.283733	11.02247
pq	1.388133	4.007361	6.305825	2.87E-10	2.60303	6.169326
MGMT	1.139027	3.123729	8.330238	8.07E-17	2.389379	4.083773
Risk	−1.89371	0.150512	−11.5915	4.55E-31	0.109272	0.207316
Risk score	0.051468	1.052815	16.8548	9.67E-64	1.046533	1.059135

Calibration analysis revealed that 1-, 3-, and 5-year were significantly contiguous to the ideal 45-degree calibration line, suggesting that the nomogram had considerable high accuracy (**Figure 7G**). Then we evaluated Concordance indices (C-index) of the three models, and the nomogram had the highest goodness of fit (0.865, **Figure 7H**). Moreover, we also compared the three different models, and 1-year ROC revealed that the nomogram exhibited the most potent predictive capacity with AUC of 0.937 (**Figure 7I**).

## Discussion

Gliomas are among the most common types of primary tumors of the central nervous system, and aberrant DNA methylation is considered the hallmark of cancer tissues, participating in carcinogenesis, tumor immunology, and recurrence (27). Due to the contribution of the fast-growing methylation bead chip technique, it is important to obtain DNA methylation profiles.

DNA methylation in vertebrates happens at position 5′ of the cytosine ring in CpGs through a covalent obligation of methyl gathering (28, 29). The loss in DNA methylation, combing the silenced tumor suppressor genes, is considered a dangerous hallmark and poor prognosis in most cancer types (30–33). Our findings are in tandem with those of previous studies. After clustering, the average methylation level of cluster1 was significantly lower than that of cluster2, indicating that cluster1 exhibited a poor prognosis. Kaplan-Meier survival analysis confirmed this idea because the overall survival outcomes of cluster1 were impressively lower than cluster2.

Another important finding was the methylation scope of immune infiltration. We postulated that cluster2 exhibited immunity and living conditions. All seven immune cells showed a significant difference, and cluster2 exhibited a higher enrichment score of B cells, NK cells, CD4T cells, and eosinocytes. In contrast, cluster1 exhibited a higher proportion of CD8+T cells, monocytes, and neutrophils. This finding is in tandem with those of the work of other studies. For example, a higher proportion of B cells is associated with increased immunity, thereby enhancing the antitumor effect leading to better prognostic outcomes (34). NK cells are the prototype innate lymphoid cells that fight against microbial contamination and tumors (35). CD4+T cells can secrete IFN-γ, IL-2, and TNFα cytokines to interfere with tumor development. Cluster2 was found to have a higher immune score and an ideal OS time, which validated the previous findings. Meanwhile, functional annotations also supported our idea since DMPs were enriched in TGF-β, IL-2, TNF-α, NK cells, and B cell receptors.

Epigenetic age acceleration is a new marker for cancer prognosis. We calculated the epigenetic age of each patient. Although the average DNAm age of cluster2 was still lower than that of cluster1, more patients in cluster1 had lower DNAm age than their chronological age. This finding is in concordance with that from earlier studies that decelerated DNAm age may result in poorer prognosis (36, 37). The strong correlation between methylation age and veritable age reveals the stability of the prediction.

We performed an integrative analysis of the TCGA Pan-cancer tumors based on other 31 cancer types. Eventually, eight cancer types were selected. Clustering based on immune-related methylation probes revealed an important common role of the immune markers, and the exact immune pattern and pathways need further evaluation. Moreover, we tested the signature on other cancers, and the seven cancer types showed affable results. One unanticipated finding was that LIHC was not concluded in clustering, but was well-distributed when tested using the signature. In the meantime, LAML and KIRP were excluded when tested using the signature. We hypothesized that there is a general DNA methylation pattern among these cancers. Thus, more studies are recommended to test this hypothesis.

Univariate Cox regression, Random Forest, and PCA were performed to construct the best prognostic signatures. To elucidate on the five probes’ biological roles, we performed GO and GSEA analysis, and the results were highly associated with immune system processes. In detail, four negative coefficient probes (gene) were cg04314652 (NFAT5), cg15734706 (RARG), cg20332503 (ZYX), and cg08985333 (CIITA). We revealed the impact of DNA methylation on gene expression in glioma, and three genes exhibited hypomethylation-upregulated DNA methylation patterns. Immunological processes of the five genes were identified to establish the signature mechanism. Nuclear factor of activated T cell 5 (NFAT5) is involved in neuroinflammation (38), and NFAT5 levels correspond to glioma pathological grade (39). Retinoic acid receptor γ (RARG) belongs to the nuclear receptor superfamily (40), and elevated RARG levels may contribute to an unfavorable outcome in LAML (41). Zyxin (ZYX) has been shown to enhance the invasion of hepatocellular carcinoma (42) and oral squamous cell carcinoma cells (43). Overexpression of ZYX is also involved in invasion and unfavorable prognosis of GBM (44). CIITA, a key regulator of the controlling major histocompatibility complex (MHCII) gene, is regulated by NFAT5 (45). The only positive coefficient probe was cg11701471 (OPRK1). In previous studies, κ-opioid receptors 1 (OPRK1) were shown to suppress lung cancer growth, suggesting a tumor-suppressive gene (46, 47). In summary, all the negative coefficient probes are tumor-genesis, and the positive one is tumor-suppressive. Since a higher risk score indicates poor prognosis, it can be hypothesized that DNA methylation silences its corresponding gene expression by hypermethylating CpG islands, shore, and shelf in the promoter regions or gene body. Previous studies of CIITA and NFAT5 strengthen the reliability of the signature.

We validated the prognostic signature using five GEO databases and the TCGA cohort itself. The risk score was associated with overall OS and PFS time. Moreover, low-risk GBM patients were more sensitive to radiation therapy. A possible explanation might be that radiation therapy activates more antitumor immune cells in higher immune cohorts, such as regulating dendritic cells (DC). Regulated DC recognizes and phagocytoses tumor cells and induces the release of inflammatory factors such as IFN-γ, IL-2, and TNFα from immune cells. Another interesting finding was that LGG patients enjoyed even worse overall survival time after radiotherapy. The benefit of radiotherapy in patients with LGG has long been controversial, and the EORTC 22845 randomized trial showed that median overall survival was similar between the radiotherapy and control groups (48). Combined with our findings, high-dose radiotherapy may lead to contrary effects such as neurotoxicity during the management of LGG patients. In the GSE48462 dataset, we found that in this LGG cohort, 14 (28%) high-risk patients benefited from RT/PCV, while no low-risk patients benefited from RT/PCV, suggesting further studies are needed to evaluate the value of PCV treatment in LGG.

Prediction of prognosis based on epigenetic change is not comprehensive. First, we confirmed that the risk score was a reliable predictor of survival through DCA. We further constructed a decision tree model to improve the risk stratification accuracy. Several factors were put into the machine learning algorithm, and the risk score was selected as a major factor, while age was selected as the secondary factor. The maneuverable decision tree helps clinicians to conveniently evaluate the patient risks. Moreover, we developed a complex nomogram model for a more accurate risk and survival outcome assessment. The nomogram model exhibited the highest concordance index when compared to other models and factors. It also exhibited the highest accuracy when compared to the decision tree and risk score model.

One limitation of our study is that bioinformatic methods were used for all analyses. Given the complicated DNA methylation pattern and intricate immune process, more experiments are needed and there exists abundant room for further progress in determining the pan-cancer immune pattern.

## Data Availability Statement

The datasets presented in this study can be found in online repositories. The names of the repository/repositories and accession number(s) can be found in the article/**Supplementary Material**.

## Author Contributions

ZH, HZ, FF, QC, ZW, YH, ZL, and XZ designed and drafted the manuscript. ZH, HZ, QC, ZD, HC, FF, JX, and ZL wrote figure legends and revised the manuscript. ZH, HZ, JX, and XZ conducted data analysis. All authors contributed to the article and approved the submitted version.

## Funding

This work was supported by the National Natural Science Foundation of China (Nos. 82073893, 81703622, 81472693, and 81873635), China Postdoctoral Science Foundation (No. 2018M633002), Hunan Provincial Natural Science Foundation of China (No. 2018JJ3838, 2018SK2101), and the Hunan Provincial Health and Health Committee Foundation of China (C2019186).

## Conflict of Interest

The authors declare that the research was conducted in the absence of any commercial or financial relationships that could be construed as a potential conflict of interest.

## Publisher’s Note

All claims expressed in this article are solely those of the authors and do not necessarily represent those of their affiliated organizations, or those of the publisher, the editors and the reviewers. Any product that may be evaluated in this article, or claim that may be made by its manufacturer, is not guaranteed or endorsed by the publisher.

## Abbreviations

AUC, Area Under Curve; CNS, central nervous system; C-index, concordance index; CDF, cumulative distribution function; DC, dendritic cells; DCA, decision curve analysis; DEGs, differentially expressed genes; DMPs, differentially methylation probes; ebGSEA, empirical Bayes Gene Set Enrichment Analysis; GBM, glioblastoma; GEO, Gene Expression Omnibus; GO, gene ontology; GSEA, gene set enrichment analysis; IL, interleukin; LGG, lower-grade glioma; MHC, major histocompatibility complex; NFAT5, Nuclear factor of activated T cells 5; NK, natural killer; OPRK1, κ-opioid receptors 1; OS, Overall Survival; PAC, Proportion of Ambiguous Clustering; PCA, principal component analysis; PFS, Progression-Free Survival; RT/PCV, radiotherapy and Procarbazine, CCNU, Vincristine; ROC, receiver operating characteristic; TGF-β, tumor growth factor-β; TNFα, tumor necrosis factor-α; SNP, single-nucleotide polymorphism; TCGA, The Cancer Genome Atlas; TME, tumor microenvironment; TNFα, tumor necrosis factor α; ZYX, zyxin.
