# Supplementary material for: Identification of Methylation Immune Subtypes and Establishment of a Prognostic Signature for Gliomas Using Immune-Related Genes
Source: Front Immunol. 2021 Nov 4;12:737650. doi: 10.3389/fimmu.2021.737650 (PMC8600480; doi:10.3389/fimmu.2021.737650)
Supplement: Supplementary file 1 [file DataSheet_1.pdf]

Supplemental Figures

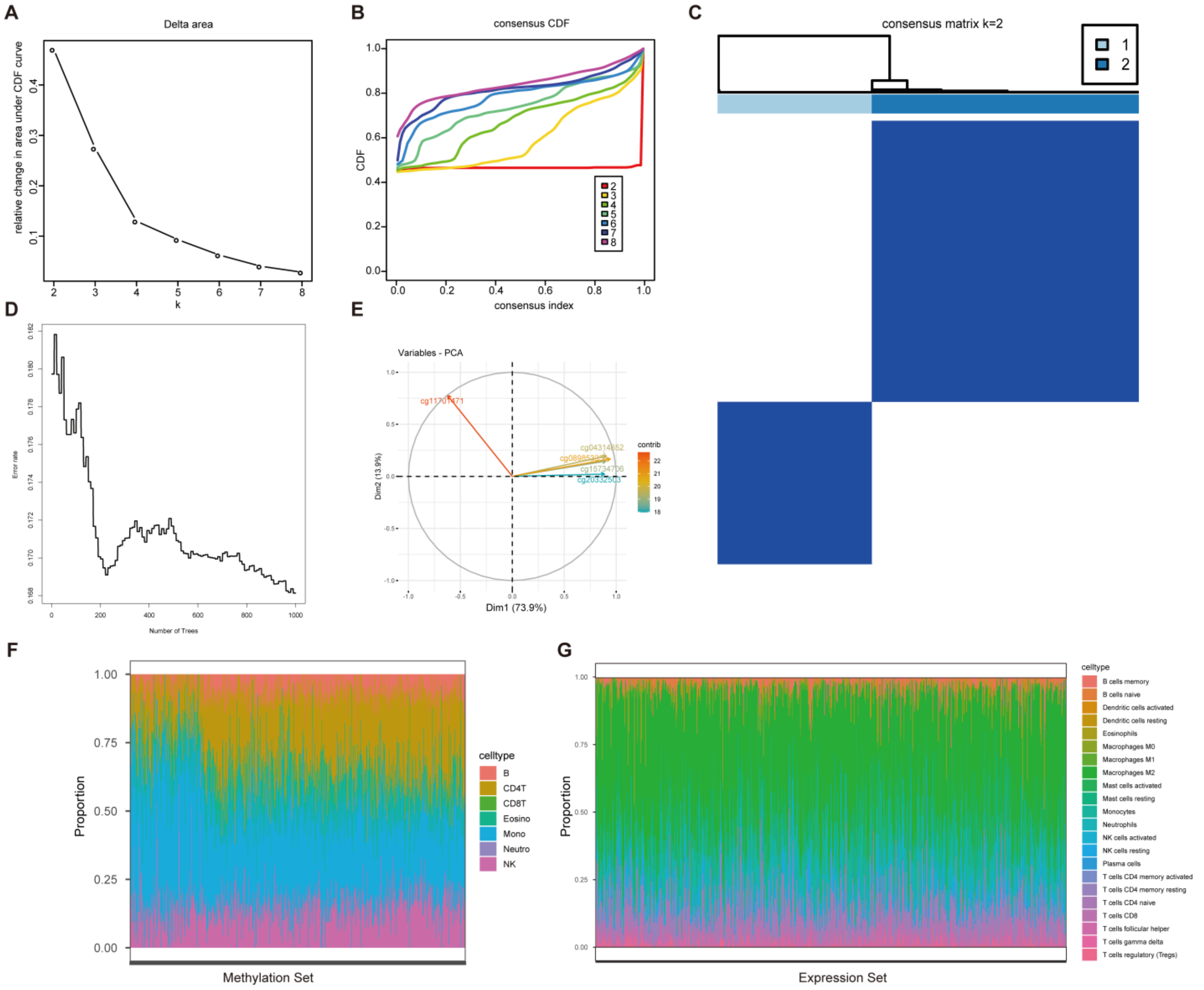

Supplemental Figure 1 (A), Delta area plot of clusters showing the relative change in area under cumulative distribution function (CDF) curve. (B), CDF plot showing consensus distributions for each k. (C), Heatmap of consensus matrix presenting each consensus score when k=2. (D), Error rate of random survival forest analysis. (E), PCA analysis with the five chosen probes. (F), Proportion of immune-infiltrating cell types in the TCGA methylation dataset. (G), Proportion of immune-infiltrating cell types in the TCGA RNA-seq cohort.

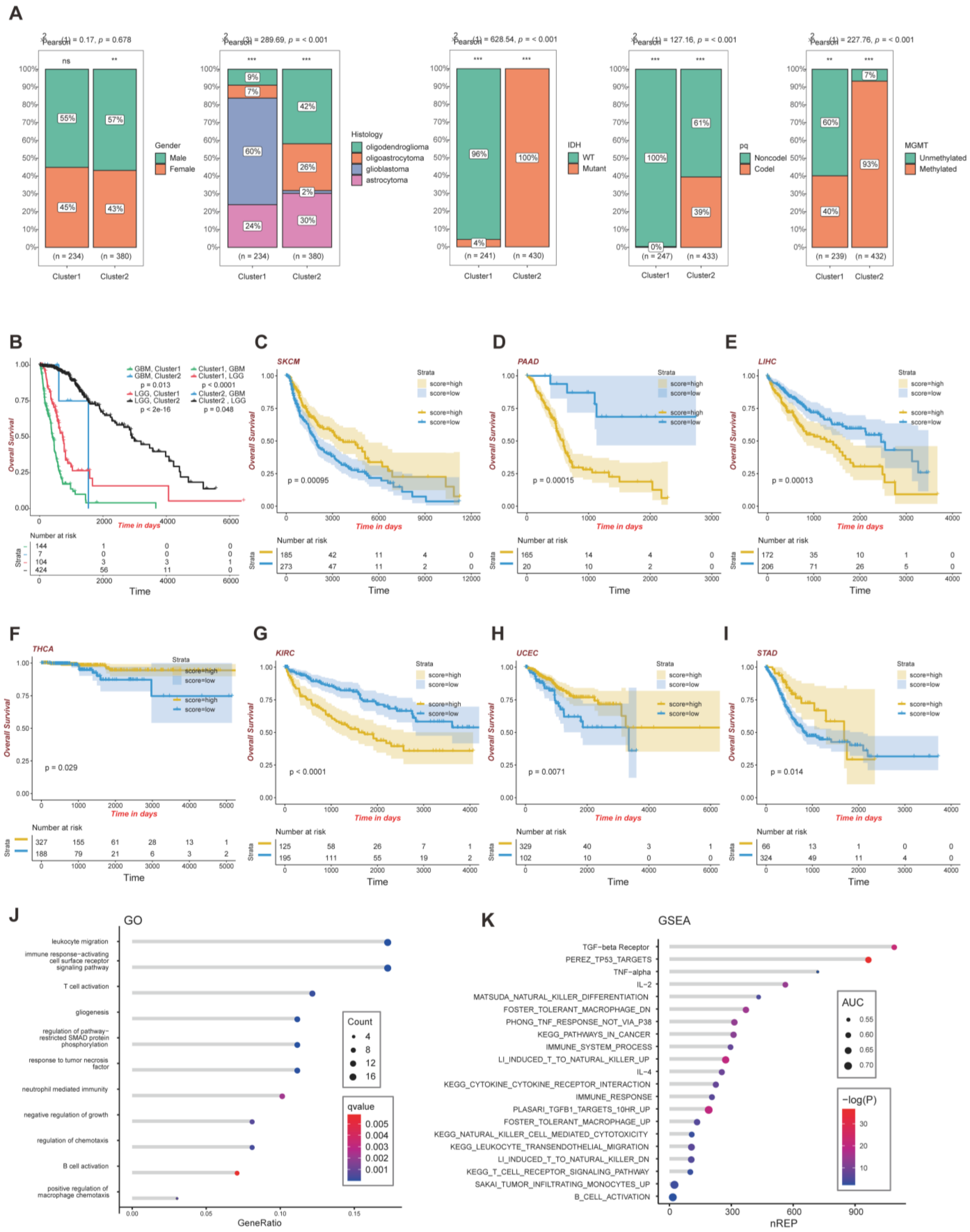

Supplemental Figure 2 (A), Discrepancy of clinical information between the two clusters. (B), Kaplan-Meier survival curves of 2 clusters in the GBM and LGG cohort. (C) to (I), Seven types of cancer showing significance in the TCGA pan-cancer survival analysis using the prognostic signature. SKCM, Skin Cutaneous Melanoma; PAAD, Pancreatic adenocarcinoma; LIHC, Liver hepatocellular carcinoma; THCA, Thyroid carcinoma; KIRC, Kidney renal clear cell carcinoma; UCEC, Uterine Corpus Endometrial Carcinoma; STAD, Stomach adenocarcinoma. (J), GO functional enrichment analysis of the high-risk and low-risk group. (K), ebGSEA result of the high-risk and low-risk group. NS, not significant; ** p<0.01; *** p<0.001.

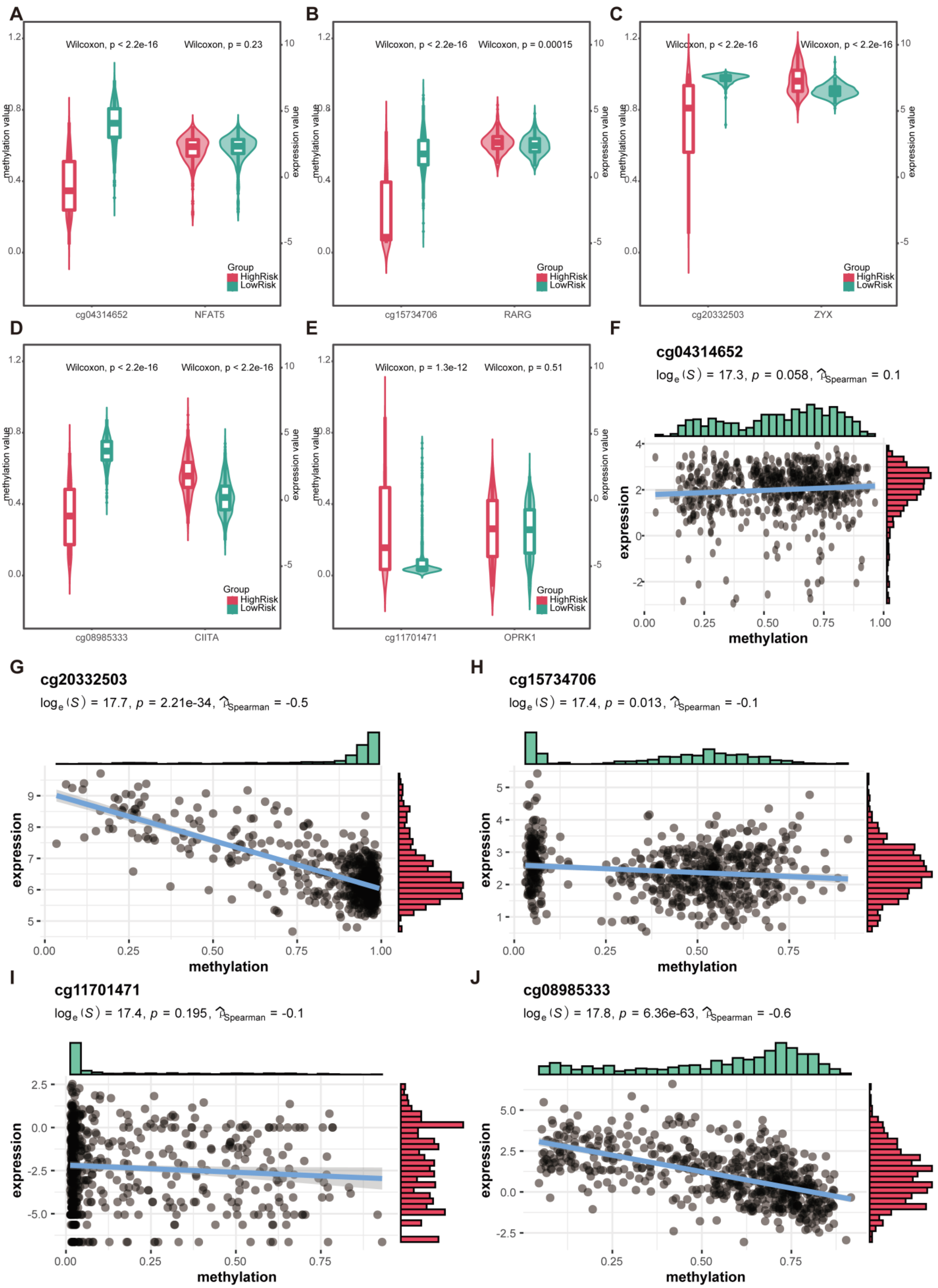

Supplemental Figure 3 The relationship between methylation and expression level of five selected probes. (A) to (E), Violin plots of DNA methylation level and the corresponding expression level. (F) to (J), Correlation between DNA methylation and gene expression.
