# Supplementary material for: Identification of Methylation Immune Subtypes and Establishment of a Prognostic Signature for Gliomas Using Immune-Related Genes
Source: Front Immunol. 2021 Nov 4;12:737650. doi: 10.3389/fimmu.2021.737650 (PMC8600480; doi:10.3389/fimmu.2021.737650)
Supplement: Supplementary file 2 [file DataSheet_2.pdf]

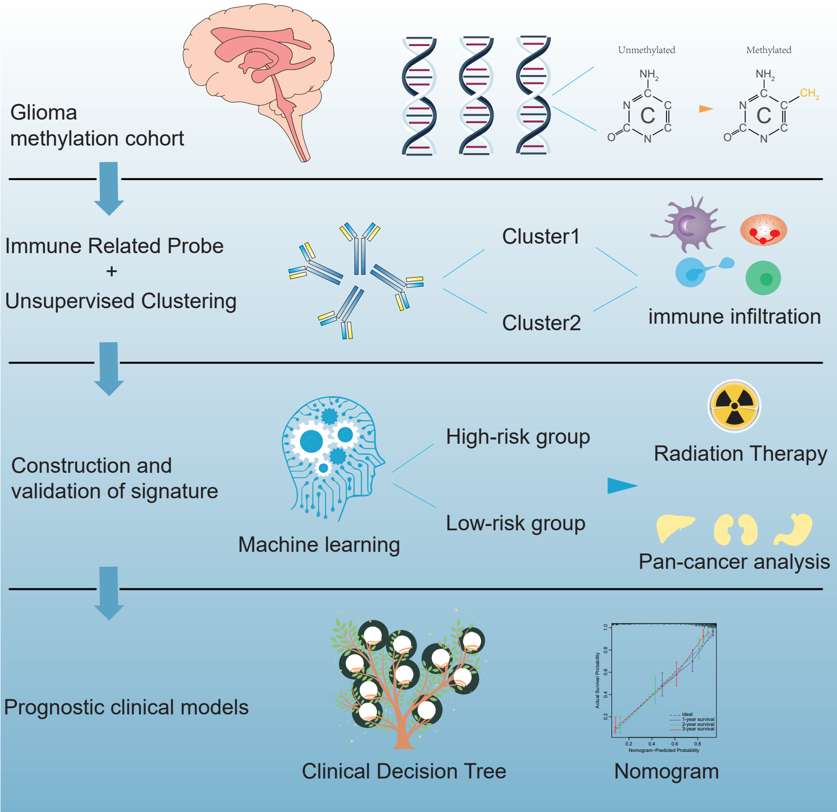

Glioma
methylation cohort
Unmethylated
Methylated
NH_2
CH_3
Immune Related Probe
+
Unsupervised Clustering
Cluster1
Cluster2
immune infiltration
Construction and
validation of signature
Machine learning
High-risk group
Low-risk group
Radiation Therapy
Pan-cancer analysis
Prognostic clinical models
Clinical Decision Tree
Nomogram
Actual Survival Probability
Nomogram−Predicted Probability
ideal
1-year survival
2-year survival
3-year survival
